# Influence of Dll4 via HIF-1α-VEGF Signaling on the Angiogenesis of Choroidal Neovascularization under Hypoxic Conditions

**DOI:** 10.1371/journal.pone.0018481

**Published:** 2011-04-19

**Authors:** Xiao Dong, Yu-Sheng Wang, Guo-Rui Dou, Hui-Yuan Hou, Yuan-Yuan Shi, Rui Zhang, Ke Ma, Lin Wu, Li-Bo Yao, Yan Cai, Jian Zhang

**Affiliations:** 1 Department of Ophthalmology, Xijing Hospital, Fourth Military Medical University, Xi'an, People's Republic of China; 2 Department of Biochemistry and Molecular Biology, State Key Laboratory of Cancer Biology, Fourth Military Medical University, Xi'an, People's Republic of China; Alcon Research, Ltd., United States of America

## Abstract

Choroidal neovascularization (CNV) is the common pathological basis of irreversible visual impairment encountered in a variety of chorioretinal diseases; the pathogenesis of its development is complicated and still imperfectly understood. Recent studies indicated that delta-like ligand 4 (Dll4), one of the Notch family ligands might participate in the HIF-1α-VEGF pathway to regulate CNV angiogenesis. But little is known about the influence and potential mechanism of Dll4/Notch signals on CNV angiogenesis. Real-time RT-PCR, Western blotting were used to analyze the expression alteration of Dll4, VEGF and HIF-1α in hypoxic RF/6A cells. Immunofluorescence staining, a laser-induced rat CNV model and intravitreal injection techniques were used to confirm the relationships among these molecules *in vitro* and *in vivo*. RPE-RF/6A cell co-culture systems were used to investigate the effects of Dll4/Notch signals on CNV angiogenesis. We found that the Dll4 was involved in hypoxia signaling in CNV angiogenesis. Results from the co-culture system showed that the enhancement of Dll4 expression in RF/6A cells led to the significantly faster proliferation and stronger tube forming ability, but inhibited cells migration and invasion across a monolayer of RPE cells in hypoxic environment, while siRNA-mediated Dll4 silencing caused the opposite effects. Pharmacological disruption of Notch signaling using gamma-secretase inhibitor (GSI) produced similar, but not identical effects, to that caused by the Dll4 siRNA. In addition, the expression of several key molecules involved in the angiogenesis of CNV was altered in RF/6A cells showing constitutively active Dll4 expression. These results suggest that Dll4 play an important role in CNV angiogenesis, which appears to be regulated by HIF-1α and VEGF during the progression of CNV under hypoxic conditions. Targeting Dll4/Notch signaling may facilitate further understanding of the mechanisms that underlie CNV angiogenesis.

## Introduction

Choroidal neovascularization (CNV), which involves both angiogenesis and vasculogenesis, occurs as the final event in a variety of chorioretinal diseases, causing severe and irreversible loss of vision. In recent decades, significant progress has been made in studies on the pathophysiologic mechanisms linked to CNV. However, the development of CNV is a complex and dynamic process, including multi-stimuli, multi-cytokines and multi-cells participate in, the specific mechanism underlying its progression is still poorly understood [1].

It has been recognized that disruption of the balance of angiogenesis promoters and inhibitors initiates CNV growth. Vascular epithelium growth factor (VEGF) is believed as the most powerful angiogenesis promoter, and played the significant role in the development and maintenance of the choroidal vasculature [2]. Pathogenesis investigation and therapy targeting VEGF in CNV have been made a lot of achievements [3]. Like VEGF, there are some molecules not only occupy an important position in embryonic vascular development, but also in adult angiogenesis. Among of them, Dll4/Notch has attracted extensive attention recently [4], [5].

Dll4 is one of the Notch ligands in mammalian cells, and is expressed specifically in the physiological and pathological vasculature [6]. Remarkably, the deletion of a single Dll4 allele resulted in early embryonic lethality due to the failure to form a functional vasculature [7], [8]. So far, only two genes, Dll4 and VEGF, are known to cause embryonic lethality due to haploinsufficiency, which emphasizes their crucial role in vascular development [9].

Flurry of publications yielded substantial insights into the important role of Dll4 in angiogenesis. During early development, Dll4 was expressed specifically in arterial endothelial cells (ECs) of the embryonic vasculature and developing retinal arteries to regulate vascular arterializations [6], [10], [11]. In the angiogenesis of the adult and tumors vascular network, Dll4 had been shown to regulate tip/stalk cell specification, and the attenuation of Dll4/Notch signaling resulted in a chaotic vascular network with excessive branching and sprouting [12]. In addition, Dll4/Notch interacted with VEGF signaling on several levels to regulate angiogenesis. It functioned downstream of VEGF signaling and its activation triggered a negative feedback loop that restrains the effects of VEGF [13], [14].

Our previous study had found that disruption of the transcription factor recombination signal-binding protein Jκ (RBP-J), the key transcription factor in Notch signaling, resulted in a more severe neovascularization at the site of laser-induced CNV. In addition, in the absence of RBP-J, proliferation of ECs increased significantly, leading to accumulative vessel outgrowth [15]. These findings suggested that Notch signaling may regulate angiogenesis of CNV by effecting on the activity of ECs. Nevertheless, the precise mechanism underlying the effects has not yet been known.

Age-related changes and stimulus underlying ocular diseases often cause hypoxia in the microenvironment surrounding the retina, Bruch's membrane and the choriocapillaris [1]. Under hypoxic conditions, retinal pigment epithelial (RPE) cells could activate hypoxia inducible factor 1α (HIF-1α) pathway to release angiogenesis promoters like VEGF [16], [17], [18], then the signaling through VEGF receptors and EphrinB2/EphB4 signal systems influenced choroidal microvascular endothelial cells (CECs) function to initiate the angiogenesis of CNV [19]. In addition, previous reports demonstrated that Dll4 was a hypoxia-regulated gene, which not only received signals from HIF-1α in ECs, but also responded to HIF-1α-VEGF signaling via the hypoxia pathway [20], [21], [22]; VEGF receptors and EphrinB2 were direct target genes regulated by Dll4 [23], [24], [25]. Combining these results, we hypothesized that Dll4/Notch signaling might participate in the HIF-1α-VEGF pathway to regulate CNV angiogenesis in pathologic retinochoroidal diseases.

In the present study, rhesus macaque RF/6A cells were employed to represent CECs, as they are convenient for experimental manipulation and share a high level of evolutionary conservation with humans. The chemical hypoxia model was established in RF/6A cells to investigate the influence of Dll4/Notch signaling on the HIF-1α-VEGF pathway and an RPE-RF/6A contacting/separating co-culture system was used to explore the role of Dll4/Notch signaling in regulating the key step in CNV angiogenesis. The results showed that Dll4 was a vascular regulator involved in CNV angiogenesis, and that it was regulated by HIF-1α and VEGF during CNV progression under hypoxic conditions. Our results provide a novel molecular mechanism for CNV and present a new potential target for CNV treatment in the future.

## Results

### Up-regulation of Dll4 expression in RF/6A cells under hypoxic condition

**Figure 1:**
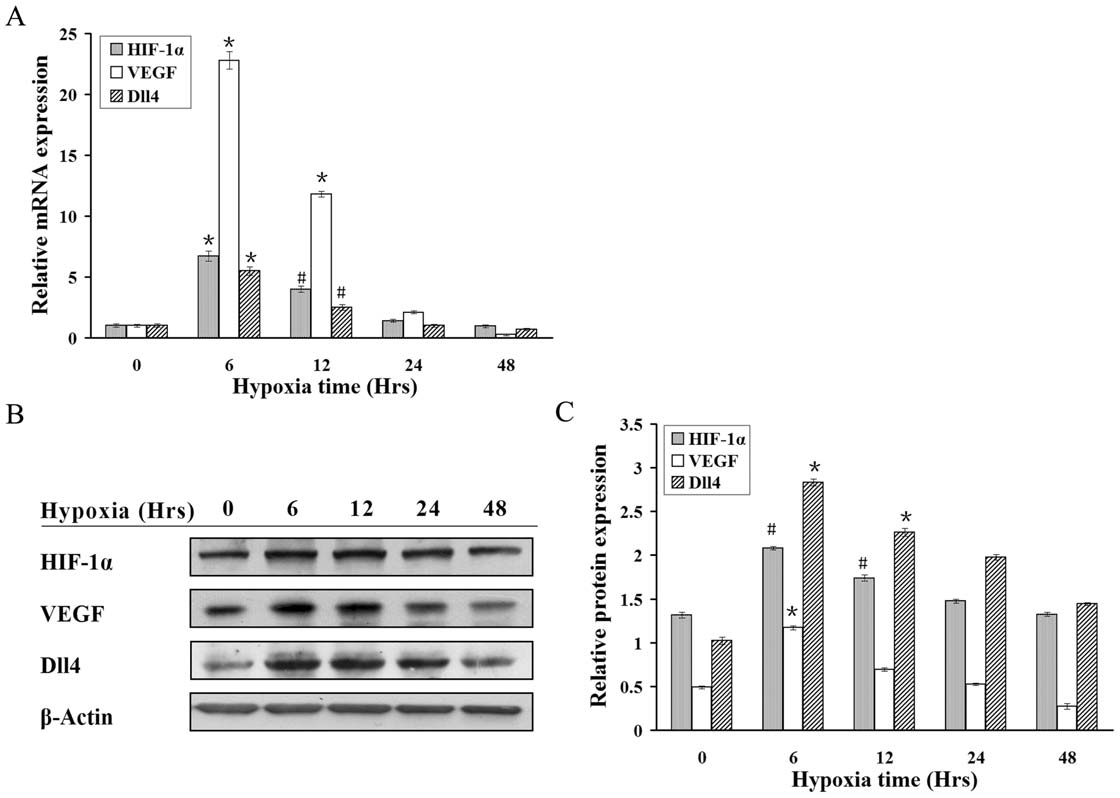
Quantitative analysis of the expression of Dll4, HIF-1α and VEGF by real-time RT-PCR and Western blotting in hypoxic RF/6A cells. Messenger RNA (A) and protein (B) expression of Dll4, HIF-1α and VEGF in RF/6A cells under hypoxia induced by 200 µM CoCl_2_ at 6, 12, 24 and 48 hrs, with 0 h as the baseline. (C) The time course of Dll4, HIF-1α and VEGF protein expression in RF/6A cells under hypoxia was consistent with the mRNA (#P<0.05, *P<0.01).

To explore the possibility that Dll4 may participate in hypoxia-mediated signaling in CNV, we investigated the effects of chemical hypoxia on Dll4 expression in RF/6A cells. As expected, real-time RT-PCR showed that Dll4 mRNA transcription increased significantly after 6 hrs of hypoxia and slowly declined thereafter (Fig. 1A). As shown by Western blotting (Fig. 1B), Dll4 protein levels in RF/6A cells were also up-regulated during hypoxia. The time course of the Dll4 protein expression profile (Fig. 1C) mirrored that for Dll4 mRNA (Fig. 1A) in hypoxic RF/6A cells. HIF-1α and VEGF expression were investigated in conjunction with Dll4 expression and the results showed that the variation in Dll4 expression was in accordance with variations in HIF-1α and VEGF levels (Fig. 1).

### Reduced expression of Dll4 and VEGF by transfection of pshHIF-1α to RF/6A cells under hypoxia

**Figure 2:**
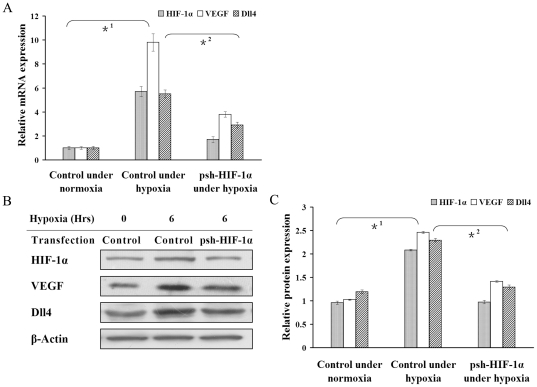
Effect of psh-HIF-1α on hypoxia-induced Dll4, HIF-1α and VEGF expression in RF/6A cells determined by real-time RT-PCR and Western blotting. Messenger RNA (A) and protein (B and C) expression of Dll4, HIF-1α and VEGF in RF/6A cells transfected with either psh-HIF-1α or control pDNA. RF/6A cells were exposed to 200 µM CoCl_2_ for 6 hrs to achieve chemical hypoxia. After 6 hrs exposure to hypoxia, Dll4, HIF-1α and VEGF expression increased in RF/6A cells transfected with control pDNA (*1 P<0.01). While transfected with psh-HIF-1a, the up-regulation of Dll4, HIF-1α and VEGF in RF/6A cells by hypoxia were abrogated (*2 P<0.01).

To confirm further whether Dll4 is regulated by HIF-1α in RF/6A cells under hypoxia, Dll4 mRNA and protein expression was measured in RF/6A cells transfected with pshHIF-1α under hypoxic conditions for 6 hrs. In pshHIF-1α-transfected RF/6A cells, the up-regulation of HIF-1α, induced by CoCl_2_, was abolished, while no effect was seen in cells transfected with the control pDNA (Fig. 2). Real-time RT-PCR results revealed that the up-regulation of Dll4 expression under hypoxia was preserved in the control transfection, but disappeared in the pshHIF-1α group (Fig. 2A). The data from the Western blotting analysis was consistent with this finding (Fig. 2B and C). At the same time, VEGF mRNA and protein expression was inhibited in a similar fashion after CoCl_2_ treatment in the pshHIF-1α-transfected RF/6A cells (Fig. 2), indicating that both Dll4 and VEGF were HIF-1α-regulated genes.

### Downstream location of Dll4 of the VEGF in CNV angiogenesis

**Figure 3:**
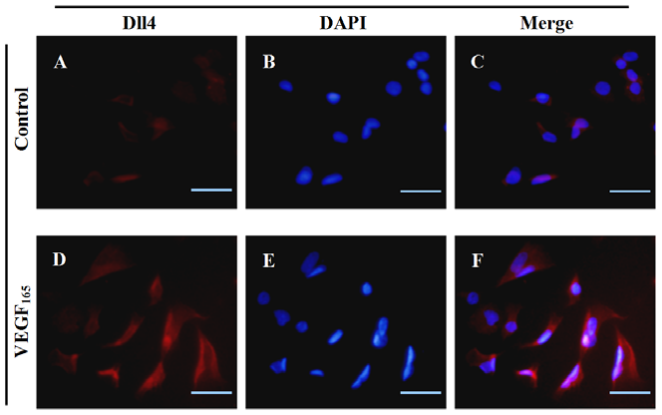
Representative immunofluorescence images of Dll4 in RF/6A cells stimulated by VEGF_165_. CY3 (red)-conjugated secondary antibody represented Dll4 expression (A and D), DAPI (blue)-stained nuclei (B and E). (C) Merged image of A and B. (F) Merged image of D and E. The results clearly showed that Dll4 expression was induced by VEGF_165_ (D–F) compared with the PBS-treated control group (A–C). Bar = 50 µm.

The next question was to determine whether Dll4 was regulated by VEGF in ocular ECs. VEGF_165_ was added exogenously to the culture medium of RF/6A cells and the resulting immunofluorescence images confirmed that Dll4 expression was induced by VEGF in RF/6A cells (Fig. 3).

**Figure 4:**
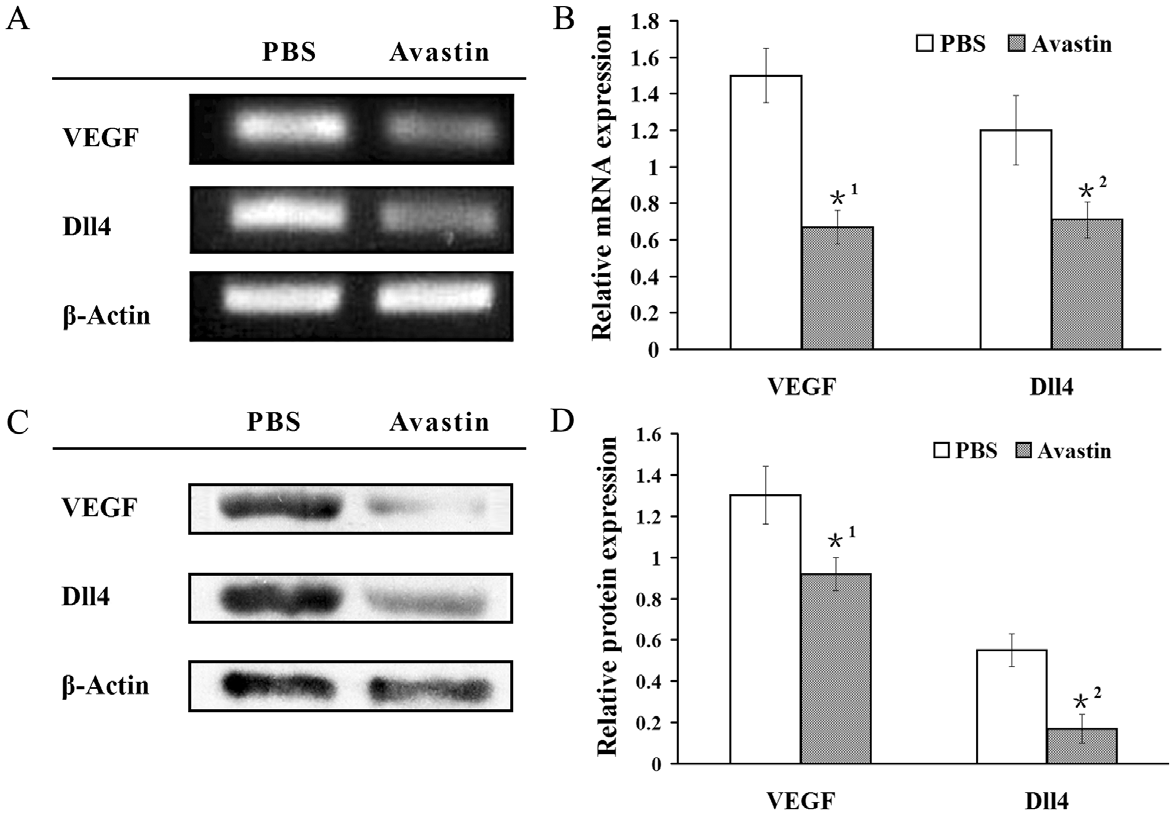
Expression of VEGF and Dll4 by intravitreal injection of Avastin in a laser-induced rat CNV model determined by RT-PCR and Western blotting. Messenger RNA expression (A and B) and protein expression (C and D) of VEGF and Dll4 in the experimental CNV model. Compared with the PBS-treated group, VEGF (*1 P<0.01) and Dll4 expression clearly decreased by intravitreal injection of Avastin.

Simultaneously, we found that the up-regulation of Dll4 after laser photocoagulation in the laser-induced rat CNV model could be inhibited by intravitreal injection of the VEGF inhibitor, Avastin (Fig. 4). These *in vivo* results further confirmed that Dll4 was located downstream of VEGF in CNV angiogenesis.

### Promotion of RF/6A cells proliferation by Dll4 in a co-culture system under hypoxia

**Figure 5:**
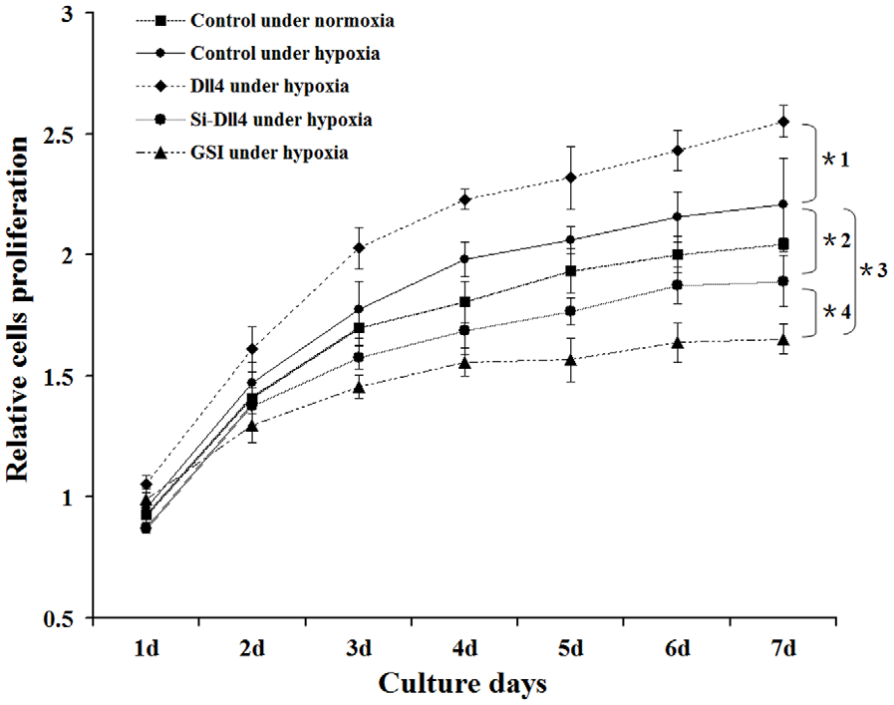
Analysis of effect of Dll4 on the proliferation of RF/6A cells co-cultured with RPE cells under hypoxia by MTT proliferation assay. RF/6A cell growth curves from five treatment groups are depicted. In the proliferation assay, hypoxia increased RF/6A cells proliferation in the co-culture system, while the up-regulation of Dll4 in RF/6A cells promoted cell growth to a remarkable extent (*1 P<0.01 vs control pDNA-transfected group under hypoxia). By contrast, cell growth was suppressed significantly by the silencing of Dll4 (*2 P<0.01) and by the total block of Notch signaling in GSI-treated cells (*3 P<0.01). Noticeably, the proliferation of GSI-treated cells was even lower than that of Si-Dll4-transfected cells (*4 P<0.01 vs Si-Dll4-transfected group under hypoxia).

Next, an RPE-RF/6A co-culture system was utilized to investigate the precise effects of Dll4, induced by RPE cells under conditions of chemical hypoxia, on RF/6A cells function. A proliferation assay showed that constitutive expression of Dll4 in RF/6A cells significantly increased cell proliferation under hypoxia in the co-culture system, compared to control pDNA-transfected cells. By contrast, cells proliferation was suppressed by silencing Dll4 expression in hypoxic RF/6A cells using the Dll4-specific siRNA, Si-D114. To confirm that the Dll4 defect in RF/6A cells was consistent with a disruption in Notch signaling, GSI treatment was used to disrupt Notch signaling pharmacologically. Interestingly, in this experiment, the suppression of cell proliferation was significantly greater than in Si-Dll4-treated cells under hypoxic conditions (Fig. 5).

### Inhibition of RF/6A cells migration by Dll4 in a co-culture system under hypoxia

**Figure 6:**
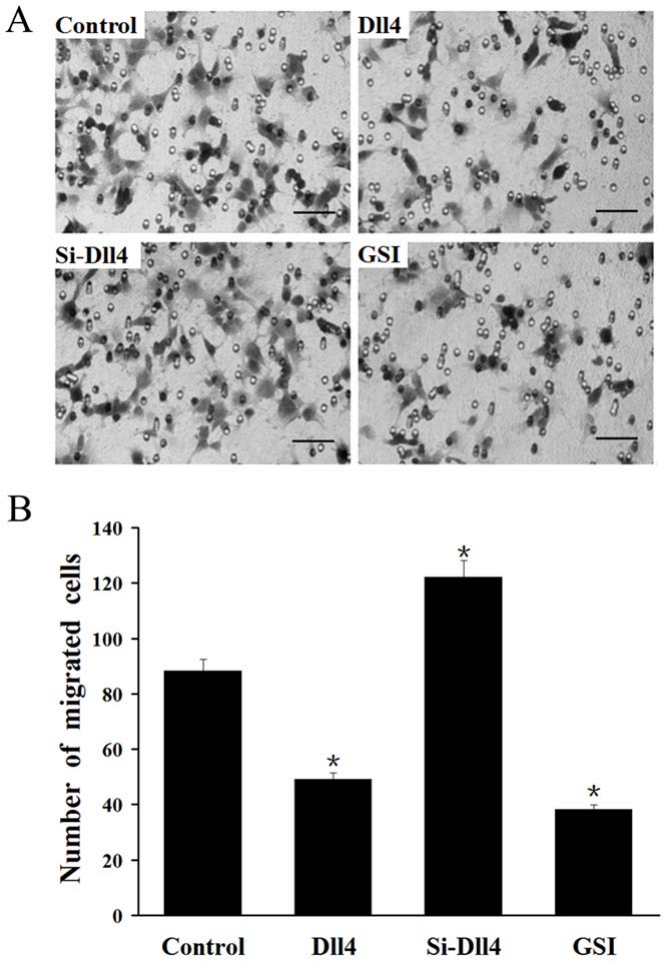
Analysis of effect of Dll4 on the migration of RF/6A cells co-cultured with RPE cells under hypoxia by transwell migration assay. (A) HE staining reveals migrated cells from each of the following groups: co-cultured RF/6A cells transfected with control pDNA group under hypoxia, Dll4 up-regulation group, Dll4 down-regulation group and GSI treated group. (B) The number of RF/6A cells that had migrated was reduced significantly by 50.6% in the group transfected with Dll4, but was increased by 41.4% in the Si-Dll4 transfected group, and was attenuated by 55.2% in the GSI treated group (* P<0.01) compared with the control pDNA group. Bar = 50 µm.

The effects of Dll4 on RF/6A cells migration in a co-culture system under hypoxia were also investigated. The number of RF/6A cells, transfected with Dll4, which migrated across the insert, was reduced by 50.6% compared with the control pDNA group, while the RPE-/hypoxia-induced RF/6A cells migration increased remarkably by 41.4% in Si-Dll4-transfected RF/6A cells. However, the blockade of Notch signaling via GSI treatment attenuated cells migration by 55.2% (Fig. 6).

### Promotion of RF/6A cells tube formation by Dll4 in a co-culture system under hypoxia

**Figure 7:**
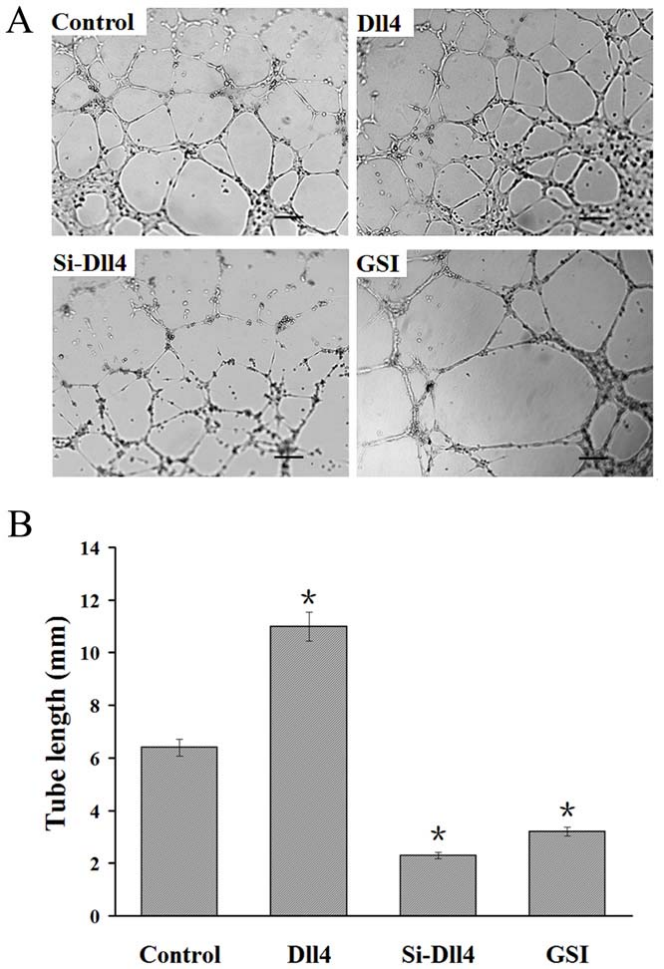
Analysis of effect of Dll4 on tube formation of RF/6A cells co-cultured with RPE cells under hypoxia by tube formation assay. (A) Microscopic images showing tube formation in each of the four treatment groups: co-cultured RF/6A cells transfected with control pDNA under hypoxia, Dll4 up-regulation group, Dll4 down-regulation group and GSI treated group. (B) Increased tube formation was observed when co-cultured RF/6A cells were transfected with Dll4 under hypoxia (*P<0.01 vs control pDNA transfected group). By contrast, reduced tube formation was seen in co-cultured RF/6A cells with down-regulated Dll4 and in cells subjected to pharmacological disruption of Notch signaling (*P<0.01). Bar = 100 µm.

Tube formation is a very important function of ECs. RF/6A cells, transfected with Dll4, and grown in a collagen matrix gel under hypoxia showed enhanced tube formation ability. This result agreed with the proliferation assay, since the ability of ECs to form tube-like structures is partly related to cell proliferation. When Dll4 expression was inhibited or Notch signaling was blocked in RF/6A cells, tube formation was reduced significantly (Fig. 7).

### Inhibition of RF/6A cells invasion by Dll4 across the RPE monolayer under hypoxia

**Figure 8:**
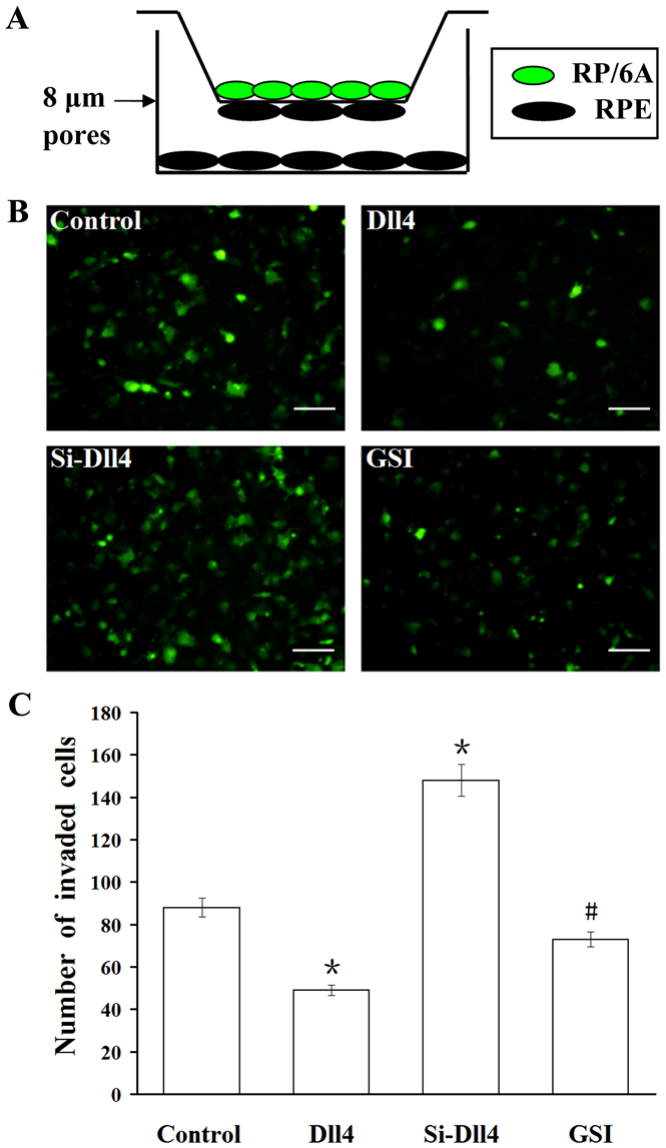
Analysis of effect of Dll4 on invasion of RF/6A cells across RPE monolayer under hypoxia in a contact co-culture system. Representative photographs of each group are shown. (A) RPE-RF/6A contact co-culture system. (B) GFP-labeled migrated RF/6A cells from each of the following groups: co-cultured cells transfected with control pDNA, Dll4 up-regulation group, Dll4 down-regulation group and GSI treated group. (C) The number of invaded RF/6A cells overexpressing Dll4 was significantly reduced compared with the control, as was the number of invaded cells in the GSI-treated group; however, the Si-Dll4 transfected group showed a noticeable increasing in invasion (# P<0.05, * P<0.01). Bar = 100 µm.

The rupture of Bruch's membrane by CECs and their migration through the RPE monolayer into the neurosensory retina is a complex and important process in the development of CNV. To determine whether Dll4 influences the invasion of RF/6A cells across an RPE monolayer, a contact co-culture system was used under hypoxic conditions to investigate the hypothesis. After GFP-labeled RF/6A cells were placed in contact with an RPE monolayer plated on the underside of the membrane for 4 hrs, the number of invaded RF/6A cells was quantified and the results showed that the number of cells that over-expressed Dll4 was significantly lower than the number of pDNA-transfected cells. The number of invaded cells was highest in the Si-Dll4 group, with slightly fewer migrated cells in the GSI treated group compared to the control (Fig. 8).

### Alterations of the expression of several critical genes by Dll4 constitutive over-expression in RF/6A cells

**Figure 9:**
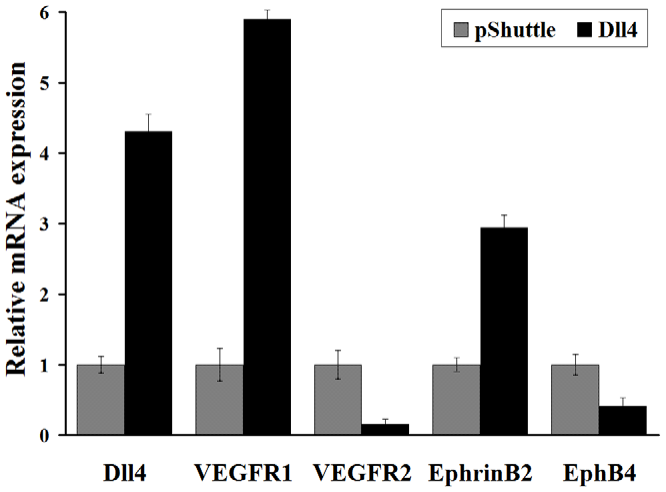
Quantitative analysis of the expression of several critical genes by real-time RT-PCR in constitutive up-regulation of Dll4 in RF/6A cells. The expression of VEGFR1 and EphrinB2 increased, but VEGFR2 and EphB4 decreased significantly in over-expression of Dll4 in RF/6A cells.

Real-time RT-PCR was used to assess further the effects of over-expressing Dll4 on key CNV angiogenesis genes downstream, including VEGFR1, VEGFR2, EphrinB2 and EphB4. Up-regulating Dll4 by 4.3-fold led to a 5.9-fold increased in VEGFR1 expression and a 2.9-fold increased in EphrinB2 expression, but an 5.7-fold decreased in VEGFR2 expression and a 1.4-fold decreased in EphB4 expression (Fig. 9). These changes in gene expression might contribute to variations in RF/6A cells behavior.

## Discussion

In the past, it has been generally accepted that the Notch pathway is an evolutionarily conserved signaling system required for normal embryonic development, the regulation of tissue homeostasis, and the maintenance of stem cells in adults [26], [27]. Notch signaling is initiated by the cleavage of the Notch intracellular domain (NICD) activated through ligand-receptor binding on cells membrane, then NICD immediately translocates to the nucleus where it interacts with transcription factors and regulates downstream genes expression, such as HES1, HES5, and HEY1 [26], [28]. Nonetheless, the essential contribution of the Notch pathway to vascular morphogenesis has been revealed only recently [4], [5], [29]. There are five transmembrane Notch ligands (Jagged1, Jagged2, Dll1, Dll3, and Dll4) and four Notch receptors (Notch1- to 4) in mammalian cells. Of note, the expressions of Dll4 and its acknowledged known receptors (Notch1 and Notch4) are relatively restricted to the vascular system, where several studies have shown that Dll4/Notch signaling is crucial [7], [8], [30], [31].

During early embryonic development, Dll4 expression was initially observed in the major arterial vessels. With embryonic maturation, this pattern of expression disappeared and became confined to the small vessels and capillaries. Consistent with the restricted expression pattern of Dll4, it had been demonstrated that Dll4/Notch signaling played a critical role in arteriovenous specification [32], acting downstream of VEGF to regulate the expression of the arterial marker EphrinB2 [24], [25]. In the studies of mouse retinal vasculature, Dll4 expression was mainly confined to the tip cells, and also endogenously distributed to the stalk cells, capillary plexus, arterial endothelium and capillary pericytes [11], [33]. Genetic and pharmacological inactivation of Dll4/Notch led to the formation of a highly branched and dense vascular network, however, the vascular structures were often not fully lumenized, generating nonproductive vessels, which indicated that both permissive and suppressive signals within the growing sprout were required for the formation of an effective vascular network [34], [35]. Therefore, Dll4 seemed to act as a caretaker, which ensured that vascularization under control, and promoted the temporal and spatial organization of a functional vasculature.

The retina is very sensitive to hypoxia. More recently it has been suggested that choroidal circulation altered in patients with age-related macular degeneration (AMD), thus resulting in a hypoxic environment surrounding the endothelium, which could change the levels of pro-angiogenic factors [36]. Retinal hypoxia stimulated HIF-1α activation and enhanced the transcription and secretion of VEGF [16]. Under hypoxic conditions, RPE cells induced the alteration of migration and proliferation of the choroidal ECs and also affected the formation of new capillary tubes [19], [37]. In this study, we have confirmed that Dll4 was a hypoxia-regulated gene, which was consistent with the previous studies [20], [21], [22]. Our data revealed that the expression of Dll4 both on mRNA and protein level significantly increased in a time-dependent manner following HIF-1α and VEGF activation responded to the chemical induced hypoxia in RF/6A cells, which was the first quantitative evaluation of the frequency and extent of Dll4 up-regulation in ocular ECs.

Several reports showed that Dll4 expression could be induced, either directly by hypoxia [20], [22], or directly in response to the release of other hypoxia-induced factors, such as VEGF [38], recapitulating it can participate in hypoxia-mediated signal pathway to regulate CNV. Consistent with this hypothesis, our data showed the hypoxic regulation of Dll4 was dependent on HIF-1α. When HIF-1α was silenced by HIF-1α shRNA transfection in RF/6A cells, the up-regulation of Dll4 and VEGF induced by CoCl_2_ were both abolished. Next, to test whether Dll4 expression depended on VEGF signaling, soluble VEGF_165_ was added to the culture medium of RF/6A cells. The results verified that Dll4 expression did, indeed, increase following VEGF stimulation. To confirm that the up-regulation of Dll4 was still required VEGF signaling, the CNV model *in vivo* was used. The data revealed that intravitreous injection of Avastin in rat CNV model, not only inhibited VEGF ocular expression, but also suppressed the mRNA and protein expression of Dll4, as shown in a previous study [38], implying that VEGF acted as the upstream of Dll4 in CNV angiogenesis. It had also been reported that the up-regulation of Dll4 by VEGF was mediated by both VEGFR1 and VEGFR2, which was dependent on phosphatidylinositol 3-kinase/Akt pathway but not MAPK/ERK or src kinases [39]. Notably, VEGF induction of Dll4 was demonstrated in the mouse retina recently [38]. Injection of VEGF_164_ into the vitreous increased expression of Dll4 in retinas, whereas injection of the VEGF antagonist VEGF-Trap reduced the expression of Dll4.

The current data provided evidence that Dll4/Notch signaling might play important role in the HIF-1α-VEGF pathway to regulate the progression of CNV under hypoxic conditions. Therefore, we investigated the exact effects of Dll4 in CNV angiogenesis. Different co-culture systems were utilized to observe the effects of RPE cells on CECs under CoCl_2_ treated hypoxia. Our findings showed that up-regulation of Dll4 expression in RF/6A cells resulted in a significant increase in proliferation and tube formation, but inhibited cell migration, while the silencing of Dll4 had the opposite effect. It was previously shown that up- or down-regulation of Dll4 in different ECs lines reduced cell proliferation, migration, and tube-like formation [22], [40], [41]. Thus, the effects of Notch signaling seemed to depend on species, microenvironment and specific cell type. During the process of CNV development, CECs not only migrate towards the chemotactic gradients produced by RPE cells, but also invade across the RPE monolayer at a later stage. It has been reported that CEC-RPE contact-induced disruption of RPE barrier properties occurred in CNV. In this situation, CECs migrated through Bruch's membrane and came into contact with the RPE, leading to further exacerbation of the already compromised blood-retinal barrier [42], [43]. However, little is known about the interactions between RPE cells and CECs and the signaling events that lead to CECs transmigration. The co-culture contact results reported in this study showed that Dll4 inhibited the invasion of RF/6A cells across the RPE monolayer. According to the results, Dll4/Notch signaling might involve in CNV angiogenesis.

In addition, whenever we pharmacologically disrupted the Notch signaling using GSI, the proliferation, tube formation, migration, and even invasion across RPE monolayer of RF/6A cells were inhibited, indicating that Notch signaling promote the procession of CNV angiogenesis. It is very noticed that the inhibition effect on CNV angiogenesis by GSI was not identical to what caused by Dll4 siRNA. These results indicated that the effect of Notch signaling on CNV angiogenesis was not only through Dll4 ligand pathway, although Dll4 was the most studied vascular regulator in Notch family. Researchers previously reported the important differences in the cellular distribution of Notch ligands during the vascular development of the retina [11]. For example, Jagged1, another critical component in the process of tip cell selection, which antagonizes Dll4/Notch signaling during angiogenesis, was detected in stalk cells, where Dll4 was absent [44]. These findings indicated distinct roles for Notch signaling during the angiogenic process of CNV.

Furthermore, we found that constructive expression of Dll4 in RF/6A cells altered the transcription of several important genes that regulate angiogenesis. Real-time RT-PCR revealed that the expression of the arterial marker genes, EphrinB2 and VEGFR1, was up-regulated in Dll4-transfected RF/6A cells, while the expression of VEGFR2 and EphB4 was down-regulated. These results indicated that the functional alteration of RF/6A cells might be due to the diverse effects of Dll4/Notch signaling on different vascular genes [19]. Consistent with our findings, other groups have verified that Dll4/Notch signaling acts in a negative feedback loop with VEGF since Notch signaling represses transcription of the VEGFR2 gene through upregulation of its target gene, HESR1 (HEY1) [23], [38], [45]. The feedback effect between Dll4 and VEGF appeared vitally important for the selection of tip-stalk cells, a key step in angiogenesis, which occurred during the angiogenic expansion, in which vascular sprouts were guided by the migration of tip cells in response to the graded distributions of matrix-bound VEGF.

CNV represents one of the most important pathological mechanisms in a series of chorioretinal diseases causing severe and irreversible visual loss, like AMD. However, the treatment of CNV was depressed by its complicated and poorly understood pathogenesis. Until now, the main focus of drug target development for AMD was the inhibition of VEGF, due to its key role in CNV. Although the early success of anti-VEGF therapy for neovascular AMD was certainly encouraging, the effects of long-term VEGF inhibition will require close monitoring. In addition, anti-VEGF therapy alone appears to be ineffective in most cases [3], additional factors or pathways that drive angiogenesis directly, or switch on at certain stages to regulate angiogenesis, need to be pursued. In our study, we have demonstrated that Dll4/Notch signaling played a role in CNV angiogenesis, which appears to be regulated by HIF-1α-VEGF signaling during the progression of CNV under hypoxic conditions. Although the contribution of Notch signaling to vascular homeostasis is complicated and remains yet to be fully understood, our findings may facilitate further understanding of the mechanisms that underlie CNV angiogenesis and provide an innovative treatment strategy for ocular angiogenesis.

## Materials and Methods

### Cell culture and chemical hypoxia model

The Rhesus macaque choroid-retinal EC line, RF/6A, was obtained from the cell bank at the Chinese Academy of Science (CAS) and cultured according to their directions and guidance. RPE cells were obtained from a mature cell line preserved in our laboratory, and the cells were cultured as described previously [46]. For the hypoxia treatment, 200 µM CoCl_2_ was added to the culture medium, and cells were harvested after 6, 12, 24 and 48 hrs. Data from cells cultured under normoxic conditions were regarded as the baseline and were defined as hypoxia o h.

### In vitro transfection

RF/6A cells (1.0×10^6^ per well) were plated in six-well plates (Corning, Costar, US). After an overnight incubation in Dulbecco's modified Eagle's Medium (DMEM), containing 10% fetal bovine serum (FBS) without antibiotics, transfection of the following constructs: a plasmid exhibiting constitutive expression of human full length Dll4 (pShuttle-CMV-hDll4) and its control plasmid, pShuttle-CMV (kind gifts from professor HanHua); a plasmid containing HIF-1α short hairpin RNA (shRNA) (Genechem, Shanghai, China, previously described in detail [19]), pshHIF-1α, and pcDNA3.1+ transcribing as non-related sequence to pshHIF-1α; Dll4 siRNA, Si-Dll4 (F: gugggucagaacugguuauTG, R: auaaccaguucugacccacCC, Biomics, US), was performed using Lipofectamine 2000 (Invitrogen, Carlsbad, CA, USA) according to the manufacturer's instructions. In brief, for 6-well plates, 2.5 µg pDNA was mixed with 5 µl Lipofectamine 2000 at a final concentration of 1.3 µg pDNA/ml, dissolved in DMEM without serum, the resulting complex was added to the cells, which were then incubated for 4 to 6 hrs. Next, the cells were washed with DMEM and incubated in DMEM with 10% FBS for a further 24 hrs until used. The amounts of siRNA and transfection agent used were optimized, and over 80% transfection efficiency was observed, using fluorescence microscopy, with little to no toxicity visible under a light microscope and optimal conditions.

### Experimental CNV model and intravitreal injection

Laser-induced CNV model of Brown Norway rats, weighing 200 to 250 g each, was performed as previously described [47], [48], [49]. Briefly, recipient rats were anesthetized, and their pupils were dilated. Laser photocoagulation (532 nm wavelength, 75 µm spot size, 0.1 second duration, and 150 mW intensity) was delivered with a slit lamp and a cornea contact lens. Eight to ten laser photocoagulations were performed in each eye between the major retinal vessels of the superior retina. Only laser spots where rupture of Bruch's membrane was confirmed with a vaporization bubble without hemorrhage were considered effective and included in the study. Intravitreal injection of 10 µl of Avastin solution (25 mg/ml) per rat eye was performed immediately after CNV induction as previously described [50], [51]. Injection of 10 µl of PBS served as the control.

All the experimental procedures were conducted in accordance with the Detailed Rules for the Administration of Animal Experiments for Medical Research Purposes issued by the Ministry of Health of China, and were received ethical approval (Permit number: 11003) by the Animal Experiment Administration Committee of Fourth Military Medical University (Xi'an, P. R. China). All efforts were made to minimize animals' suffering and to reduce the number of animals used.

### Real-time and semi-quantitative RT-PCR analysis

RNA, extracted from cells with Trizol reagent (Invitrogen, Life Technologies, US), was converted to cDNA with the RevertAid™ First Strand cDNA Synthesis Kit (Fermantas, CA). Real-time PCR was performed using the ABI Prism® 7500 Real-Time PCR Detection System (ABI, US) and the SYBR® Premix Ex Taq™ II Kit (Takara, Japan) according to the manufacturer's instructions. The relative gene expression levels were calculated using the 2-ΔΔCt method, where Ct represents the threshold cycle, and GAPDH was used as a reference gene. The primer sequences are shown in Table 1.

**Table 1:** Primers for Real-time RT-PCR.

Genes	Forward primer sequence	Reverse primer sequence
*Dll4*	5′-CCCTGGCAATGTACTTGTGAT-3′	5′-TGGTGGGTGCAGTAGTTGAG-3′
*HIF-1α*	5′-TCGGCGAAGTAAAGAATCTGAA-3′	5′-CAAATCACCAGCATCCAGAAG-3′
*VEGF*	5′-CGCCTCTCCAAAAAGCTACAC-3′	5′-CTCACAGGAAACCGGACATC-3′
*VEGFR-1*	5′-CAGCATACCTCACTGTTCAAGG-3′	5′-CCACACAGGTGCATGTTAGAG-3′
*VEGFR-2*	5′-CGCGCCTCCTTCTAGACA-3′	5′-AGAAACACTAGGCAAACCCACA-3′
*EphrinB2*	5′-GAGCCCGGCGAACATTTACAT-3′	5′-TTCAGTCAATTCTCCAGCACGC-3′
*EphB4*	5′-TGGTCATTGTGGTCGCAGTTC-3′	5′-CCTCACAGCCTCATTAGGGTCTTC-3′
*GAPDH*	5′-GCGCTGAGTACGTCGTGGAG-3′	5′-CAGTTGGTGGTGCAGGAGG-3′

For the animal experiments, total RNA was prepared from four eyecups (RPE-choroid-sclera complex) from each group 7 days after photocoagulation. The PCR-amplified products were visualized via agarose gel (1%) electrophoresis and band intensities were quantitated using Kodak Digital Science one-dimensional software (Eastman Kodak Co., New Haven, CT). The intensity of the band in each lane was normalized to the intensity of β-Actin. The primer sequences used are shown in Table 2.

**Table 2:** Primers for semi-quantitative RT-PCR.

Genes	Forward primer sequence	Reverse primer sequence
*Dll4*	5′-GCTGGAAGTGGATTGTGG-3′	5′-CTTGTCGCTGTGAGGATAC-3′
*VEGF*	5′- AGCCCATGAAGTGGTGAA -3′	5′- TGCGGATCTTGGACAAAC -3′
*β-Actin*	5′-CACCCGCGAGTACAACCTTC -3′	5′-CCCATACCCACCATCACACC -3′

### Western blot analysis

RF/6A cells and rat tissue samples from the RPE-choroidal complexes were homogenized in RIPA lysis buffer and insoluble material was removed by centrifugation at 4°C. Forty micrograms of total protein extract from each sample were resolved by 10% SDS-PAGE and transferred to nitrocellulose membranes (Amersham Biosciences). The membranes were blocked in 5% milk and probed with the following antibodies: goat polyclonal anti-Delta-4 (1∶5000, Santa Cruz Biotech, Santa Cruz, CA, USA), mouse monoclonal anti-HIF-1α (1∶2000, Chemicon, Temecula, CA, USA) or mouse monoclonal anti-VEGF (1∶1500, Santa Cruz Biotech, Santa Cruz, CA, USA) overnight at 4°C. Membranes were washed and incubated with a horseradish peroxidase (HRP)-conjugated secondary antibody (Santa Cruz Biotech, Santa Cruz, CA, USA) for 1 h at 37°C. The addition of chemiluminescent HRP substrate solution (Millipore, USA) was used to develop the images.

### Immunofluorescence staining

RF/6A cells were allowed to adhere to glass coverslips at the bottom of 24-well plates for 12 hrs. For VEGF_165_ treatment, culture medium with a final concentration of 5 ng/ml of VEGF_165_ (Calbiochem, EMD Biosciences, USA) was added to wells for 6 hrs before cell fixation. After fixation and permeabilization, cells were incubated with the primary antibody, Dll4 (1∶150; Santa Cruz, US), and a CY3-conjugated secondary antibody. Specificity of staining was assessed by substitution of nonimmune serum for the primary antibody. Following the incubation steps suggested by the manufacturer, cell nuclei were stained with 40,6-diamino-2-phenylindole (DAPI; Molecular Probes, Eugene, OR). The slides were coverslipped with anti-fading medium and examined under a fluorescence microscope.

### Co-culture Assays

Three types of co-culture model, each of which maintained the natural anatomical relationship between basal RPE cells and CECs, were used for proliferation, migration and tube formation assays as previously described [19]. Briefly, for the proliferation and tube formation assay models, RPE cells were plated in Costar Transwells (Costar/Corning, NY) with 0.40 µm pore-size inserts, which were put in wells where RF/6A cells were plated. In addition, the wells for the tube formation assay were coated with 200 µl of extracellular matrix (ECM). For the migration assay, RF/6A cells were plated in Costar Transwells (Costar/Corning, NY) with 8.0 µm pore-size inserts, which were put in wells where RPE cells was plated. The specific procedures for the respective assays are described in detail below.

### Proliferation assay

To test the effects of RPE cells on RF/6A cells proliferation under hypoxic conditions, a proliferation assay model was used. RF/6A cells were plated at a density of 4.0×10^4^ cells/cm^2^ in 24-well plates in complete medium, and allowed to adhere overnight. Next, cells were transfected with Dll4, control pDNA or Si-Dll4 and treated with gamma secretase inhibitor (GSI) (EMD Biosciences, San Diego, CA, USA) before 0.40 µm pore-size inserts were placed in the wells. Medium containing 200 µM CoCl_2_ was added and was changed every three days. Cell proliferation was measured using a modified MTT (3-(4,5-dimethylthiazol-2-yl)-2,5-diphenyl tetrazolium bromide) assay on days 1, 2, 3, 4, 5, 6 and 7. Briefly, 100 µl MTT was added per well after the inserts were removed, and the RF/6A cells were incubated for 4 hrs. The formazan crystals formed were dissolved in 750 µl dimethyl sulfoxide after the medium was aspirated. The solution was transferred to a 96-well plate and the optical density value was recorded at 570 nm on a Microplate Reader (Model 680, Bio-Rad, USA). The results were expressed relative to the OD value of the RF/6A cells monoculture on day one of the assay.

### Migration assay

To test the effect of RPE cells on RF/6A cells migration under hypoxic conditions, a migration assay model was used. RPE cells were plated at density of 1.0×10^5^/cm^2^ in 24-well plates and medium containing 200 µM CoCl_2_ was added to the wells for 12 hrs. The RF/6A cell migration assay was performed using matrigel-coated, Costar Transwell inserts with an 8.0 µm pore-size. Briefly, 5.0×10^4^ RF/6A cells were seeded onto inserts and incubated with DMEM containing 1% FBS. After 1 h attachment, the inserts were transferred to 24-well plates as described above. After incubation for 4 hrs, the inserts were fixed with 4% paraformaldehyde and stained with hematoxylin and eosin. The number of migrated cells was counted using phase contrast microscopy (200×). Five randomly chosen fields were counted per insert.

### Tube formation assay

A co-culture model was used for the tube formation assay, in which two-dimensional tube formation was measured in a collagen gel. For this assay, 24-well plates were coated with 200 µl ECM (Sigma-Aldrich, St. Louis, MO, USA) and incubated at 37°C for 1 h to form gels. After polymerization of the gels, 1.0×10^5^ RF/6A cells were seeded into each well and incubated with 1.0 ml DMEM containing 1% FBS. Next, inserts were placed in the wells as described in the proliferation assay. Five different fields were chosen randomly in each well, and photographs were taken with a phase-contrast microscope in 24 hrs. The length of the tubes was measured using Image-Pro Plus software (Media Cybernetics, L.P., Silver Spring, MD, USA) and was expressed as a total length (mm) per microscopic field for each single well.

### Contact co-culture invasion assay

The RPE-CEC contact co-culture system had been described previously [43], [52]. Invasion assays were performed with 8.0 µm pore-size Costar Transwell inserts (Costar/Corning, NY) that permit cell migrate across the filter. 5.0×10^4^ RPE cells were grown for 6 hrs on the underside of the transwell inserts to form an organized monolayer. 5.0×10^4^ RF/6A cells were infected with the Ade-GFP virus for 24 hrs before plating inside the inserts and 2.0×10^5^ RPE were plated in the lower well under hypoxic conditions to provide chemoattractants. The undersides of the filters were fixed 6 hrs after the RF/6A cells were plated, and the migrated cells were counted under a fluorescence microscope.

### Statistical analysis

Experiments were performed three times, and each experiment was performed in triplicate. All data from quantitative assays were expressed as the mean±standard deviation and were analyzed statistically using one-way ANOVA analysis and the independent samples t-test. P<0.01 was considered to be statistically significant.

## References

[1] Grossniklaus HE, Green WR (2004). Choroidal neovascularization.. Am J Ophthalmol.

[2] Saint-Geniez M, Maldonado AE, D'Amore PA (2006). VEGF expression and receptor activation in the choroid during
development and in the adult.. Invest Ophthalmol Vis Sci.

[3] Penn JS, Madan A, Caldwell RB, Bartoli M, Caldwell RW (2008). Vascular endothelial growth factor in eye
disease.. Prog Retin Eye Res.

[4] Hofmann JJ, Iruela-Arispe ML (2007). Notch signaling in blood vessels: who is talking to whom about
what?. Circ Res.

[5] Phng LK, Gerhardt H (2009). Angiogenesis: a team effort coordinated by notch.. Dev Cell.

[6] Shutter JR, Scully S, Fan W, Richards WG, Kitajewski J (2000). Dll4, a novel Notch ligand expressed in arterial
endothelium.. Genes Dev.

[7] Gale NW, Dominguez MG, Noguera I, Pan L, Hughes V (2004). Haploinsufficiency of delta-like 4 ligand results in embryonic
lethality due to major defects in arterial and vascular
development.. Proc Natl Acad Sci U S A.

[8] Krebs LT, Shutter JR, Tanigaki K, Honjo T, Stark KL (2004). Haploinsufficient lethality and formation of arteriovenous
malformations in Notch pathway mutants.. Genes Dev.

[9] Ferrara N, Carver-Moore K, Chen H, Dowd M, Lu L (1996). Heterozygous embryonic lethality induced by targeted inactivation
of the VEGF gene.. Nature.

[10] Mailhos C, Modlich U, Lewis J, Harris A, Bicknell R (2001). Delta4, an endothelial specific notch ligand expressed at sites
of physiological and tumor angiogenesis.. Differentiation.

[11] Hofmann JJ, Luisa IM (2007). Notch expression patterns in the retina: An eye on
receptor-ligand distribution during angiogenesis.. Gene Expr Patterns.

[12] Suchting S, Freitas C, le Noble F, Benedito R, Breant C (2007). The Notch ligand Delta-like 4 negatively regulates endothelial
tip cell formation and vessel branching.. Proc Natl Acad Sci U S A.

[13] Li JL, Harris AL (2009). Crosstalk of VEGF and Notch pathways in tumour angiogenesis:
therapeutic implications.. Front Biosci.

[14] Jakobsson L, Bentley K, Gerhardt H (2009). VEGFRs and Notch: a dynamic collaboration in vascular
patterning.. Biochem Soc Trans.

[15] Dou GR, Wang YC, Hu XB, Hou LH, Wang CM (2008). RBP-J, the transcription factor downstream of Notch receptors, is
essential for the maintenance of vascular homeostasis in adult
mice.. FASEB J.

[16] Arjamaa O, Nikinmaa M, Salminen A, Kaarniranta K (2009). Regulatory role of HIF-1alpha in the pathogenesis of age-related
macular degeneration (AMD).. Ageing Res Rev.

[17] Zhang P, Zhang X, Hao X, Wang Y, Hui Y (2009). Rac1 activates HIF-1 in retinal pigment epithelium cells under
hypoxia.. Graefes Arch Clin Exp Ophthalmol.

[18] Zhang P, Wang Y, Hui Y, Hu D, Wang H (2007). Inhibition of VEGF expression by targeting HIF-1 alpha with small
interference RNA in human RPE cells.. Ophthalmologica.

[19] Zhao W, Wang YS, Hui YN, Zhu J, Zhang P (2008). Inhibition of proliferation, migration and tube formation of
choroidal microvascular endothelial cells by targeting HIF-1alpha with short
hairpin RNA-expressing plasmid DNA in human RPE cells in a coculture
system.. Graefes Arch Clin Exp Ophthalmol.

[20] Diez H, Fischer A, Winkler A, Hu CJ, Hatzopoulos AK (2007). Hypoxia-mediated activation of Dll4-Notch-Hey2 signaling in
endothelial progenitor cells and adoption of arterial cell
fate.. Exp Cell Res.

[21] Jubb AM, Turley H, Moeller HC, Steers G, Han C (2009). Expression of delta-like ligand 4 (Dll4) and markers of hypoxia
in colon cancer.. Br J Cancer.

[22] Patel NS, Li JL, Generali D, Poulsom R, Cranston DW (2005). Up-regulation of delta-like 4 ligand in human tumor vasculature
and the role of basal expression in endothelial cell
function.. Cancer Res.

[23] Taylor KL, Henderson AM, Hughes CC (2002). Notch activation during endothelial cell network formation in
vitro targets the basic HLH transcription factor HESR-1 and downregulates
VEGFR-2/KDR expression.. Microvasc Res.

[24] Hainaud P, Contreres JO, Villemain A, Liu LX, Plouet J (2006). The role of the vascular endothelial growth factor-Delta-like 4
ligand/Notch4-ephrin B2 cascade in tumor vessel remodeling and endothelial
cell functions.. Cancer Res.

[25] Yamanda S, Ebihara S, Asada M, Okazaki T, Niu K (2009). Role of ephrinB2 in nonproductive angiogenesis induced by
Delta-like 4 blockade.. Blood.

[26] Lai EC (2004). Notch signaling: control of cell communication and cell
fate.. Development.

[27] Artavanis-Tsakonas S, Rand MD, Lake RJ (1999). Notch signaling: cell fate control and signal integration in
development.. Science.

[28] Kopan R, Ilagan MX (2009). The canonical Notch signaling pathway: unfolding the activation
mechanism.. Cell.

[29] Shawber CJ, Kitajewski J (2004). Notch function in the vasculature: insights from zebrafish, mouse
and man.. Bioessays.

[30] Benedito R, Duarte A (2005). Expression of Dll4 during mouse embryogenesis suggests multiple
developmental roles.. Gene Expr Patterns.

[31] Leslie JD, Ariza-McNaughton L, Bermange AL, McAdow R, Johnson SL (2007). Endothelial signalling by the Notch ligand Delta-like 4 restricts
angiogenesis.. Development.

[32] Lawson ND, Scheer N, Pham VN, Kim CH, Chitnis AB (2001). Notch signaling is required for arterial-venous differentiation
during embryonic vascular development.. Development.

[33] Claxton S, Fruttiger M (2004). Periodic Delta-like 4 expression in developing retinal
arteries.. Gene Expr Patterns.

[34] Scehnet JS, Jiang W, Kumar SR, Krasnoperov V, Trindade A (2007). Inhibition of Dll4-mediated signaling induces proliferation of
immature vessels and results in poor tissue perfusion.. Blood.

[35] Noguera-Troise I, Daly C, Papadopoulos NJ, Coetzee S, Boland P (2007). Blockade of Dll4 inhibits tumour growth by promoting
non-productive angiogenesis.. Novartis Found Symp.

[36] Ottino P, Finley J, Rojo E, Ottlecz A, Lambrou GN (2004). Hypoxia activates matrix metalloproteinase expression and the
VEGF system in monkey choroid-retinal endothelial cells: Involvement of
cytosolic phospholipase A2 activity.. Mol Vis.

[37] Sakamoto T, Sakamoto H, Murphy TL, Spee C, Soriano D (1995). Vessel formation by choroidal endothelial cells in vitro is
modulated by retinal pigment epithelial cells.. Arch Ophthalmol.

[38] Lobov IB, Renard RA, Papadopoulos N, Gale NW, Thurston G (2007). Delta-like ligand 4 (Dll4) is induced by VEGF as a negative
regulator of angiogenic sprouting.. Proc Natl Acad Sci U S A.

[39] Liu ZJ, Shirakawa T, Li Y, Soma A, Oka M (2003). Regulation of Notch1 and Dll4 by vascular endothelial growth
factor in arterial endothelial cells: implications for modulating
arteriogenesis and angiogenesis.. Mol Cell Biol.

[40] Chen L, Lu W, Wei B, Wang N, Li T (2009). Influence of Delta-like ligand 4/Notch signal transduction
pathway upon the biological behavior of human umbilical vein endothelial
cells.. Zhonghua Yi Xue Za Zhi.

[41] Williams CK, Li JL, Murga M, Harris AL, Tosato G (2006). Up-regulation of the Notch ligand Delta-like 4 inhibits
VEGF-induced endothelial cell function.. Blood.

[42] Hartnett ME, Lappas A, Darland D, McColm JR, Lovejoy S (2003). Retinal pigment epithelium and endothelial cell interaction
causes retinal pigment epithelial barrier dysfunction via a soluble
VEGF-dependent mechanism.. Exp Eye Res.

[43] Peterson LJ, Wittchen ES, Geisen P, Burridge K, Hartnett ME (2007). Heterotypic RPE-choroidal endothelial cell contact increases
choroidal endothelial cell transmigration via PI 3-kinase and
Rac1.. Exp Eye Res.

[44] Benedito R, Roca C, Sorensen I, Adams S, Gossler A (2009). The notch ligands Dll4 and Jagged1 have opposing effects on
angiogenesis.. Cell.

[45] Henderson AM, Wang SJ, Taylor AC, Aitkenhead M, Hughes CC (2001). The basic helix-loop-helix transcription factor HESR1 regulates
endothelial cell tube formation.. J Biol Chem.

[46] Wang YS, Hui YN, Wiedemann P (2002). Role of apoptosis in the cytotoxic effect mediated by
daunorubicin in cultured human retinal pigment epithelial
cells.. J Ocul Pharmacol Ther.

[47] Hou HY, Liang HL, Wang YS, Zhang ZX, Wang BR (2010). A therapeutic strategy for choroidal neovascularization based on
recruitment of mesenchymal stem cells to the sites of
lesions.. Mol Ther.

[48] Yang XM, Wang YS, Zhang J, Li Y, Xu JF (2009). Role of PI3K/Akt and MEK/ERK in mediating hypoxia-induced
expression of HIF-1alpha and VEGF in laser-induced rat choroidal
neovascularization.. Invest Ophthalmol Vis Sci.

[49] Zhu J, Wang YS, Zhang J, Zhao W, Yang XM (2009). Focal adhesion kinase signaling pathway participates in the
formation of choroidal neovascularization and regulates the proliferation
and migration of choroidal microvascular endothelial cells by acting through
HIF-1 and VEGF expression in RPE cells.. Exp Eye Res.

[50] Lu F, Adelman RA (2009). Are intravitreal bevacizumab and ranibizumab effective in a rat
model of choroidal neovascularization?. Graefes Arch Clin Exp Ophthalmol.

[51] Sancho-Tello M, Johnsen-Soriano S, Muriach M, Bosch-Morell F, Diaz-Llopis M (2009). Transient bevacizumab (avastin)-induced alterations in rat
eyes.. Ophthalmic Res.

[52] Geisen P, McColm JR, Hartnett ME (2006). Choroidal endothelial cells transmigrate across the retinal
pigment epithelium but do not proliferate in response to soluble vascular
endothelial growth factor.. Exp Eye Res.

